# Supplementary figures and images for: A nonstructural protein 1 capture enzyme-linked immunosorbent assay specific for dengue viruses
Source: PLoS One. 2023 May 18;18(5):e0285878. doi: 10.1371/journal.pone.0285878 (PMC10194908; doi:10.1371/journal.pone.0285878)

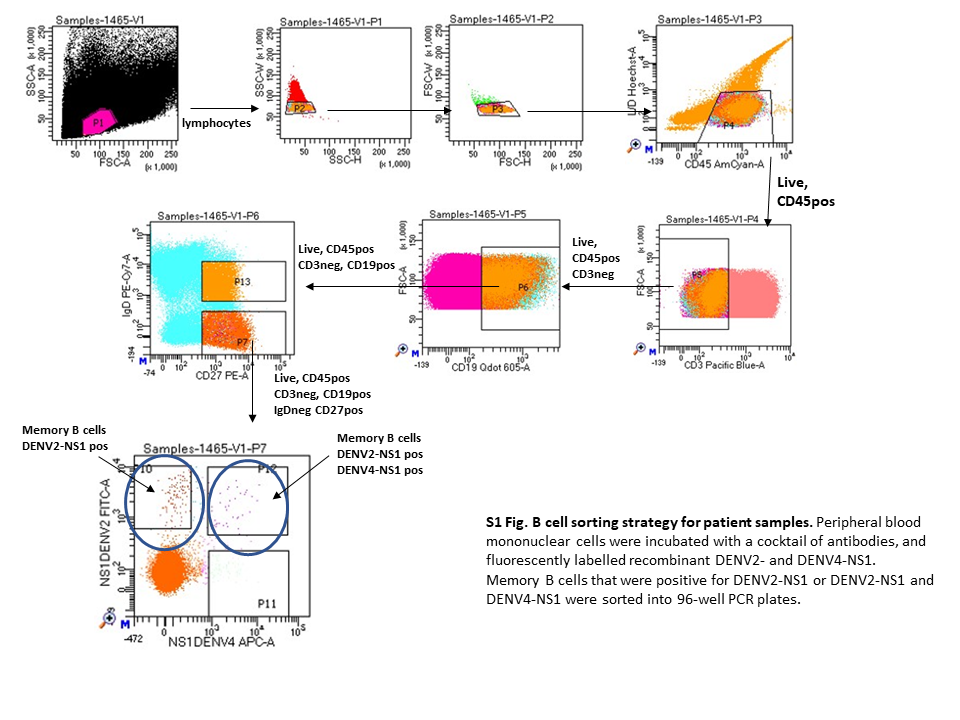

Supplement: S1 Fig — Peripheral blood mononuclear cells were incubated with a cocktail of antibodies, and fluorescently labelled recombinant DENV2- and DENV4-NS1. Memory B cells that were positive for DENV2-NS1 or DENV2-NS1 and DENV4-NS1 were sorted into 96-well PCR plates. (TIF) [file pone.0285878.s001.tif]
