# Supplementary material for: A nonstructural protein 1 capture enzyme-linked immunosorbent assay specific for dengue viruses
Source: PLoS One. 2023 May 18;18(5):e0285878. doi: 10.1371/journal.pone.0285878 (PMC10194908; doi:10.1371/journal.pone.0285878)
Supplement: S1 Table — (DOCX) [file pone.0285878.s002.docx]

Supplementary table 1. Nucleotide and amino acid sequences of the CDR of heavy and light chains of A2, D6, and D8.

**A2 antibody**

| **Heavy chain** |
| --- |
| 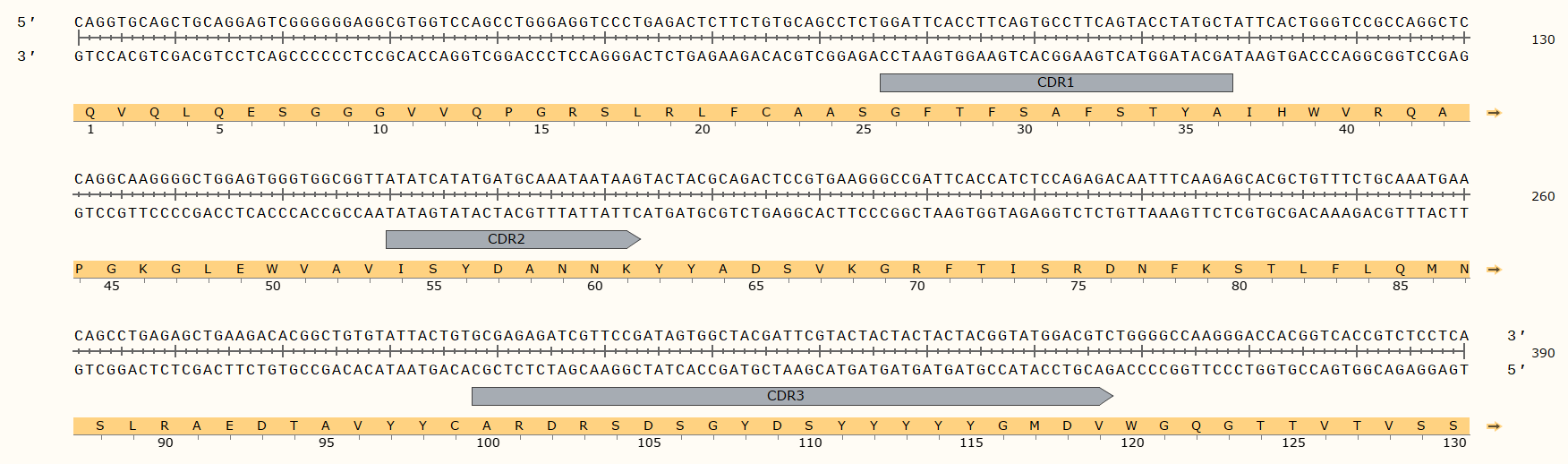 |
|  |
| **Light Chain** |
| **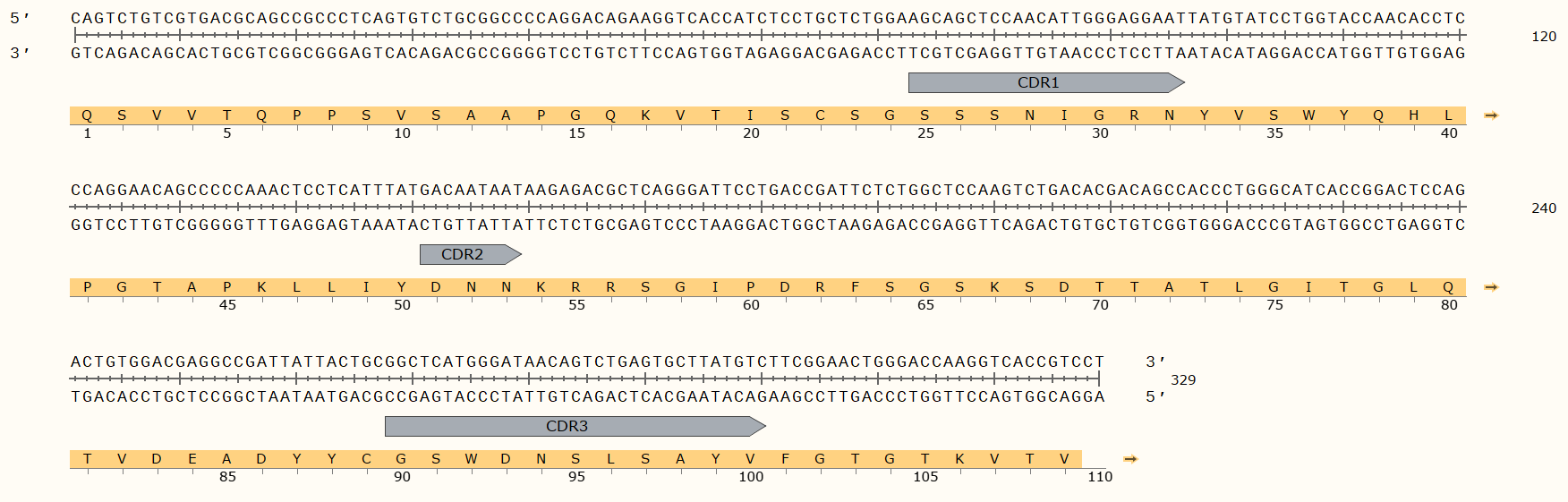** |

**D6 antibody**

| **Heavy chain** |
| --- |
| **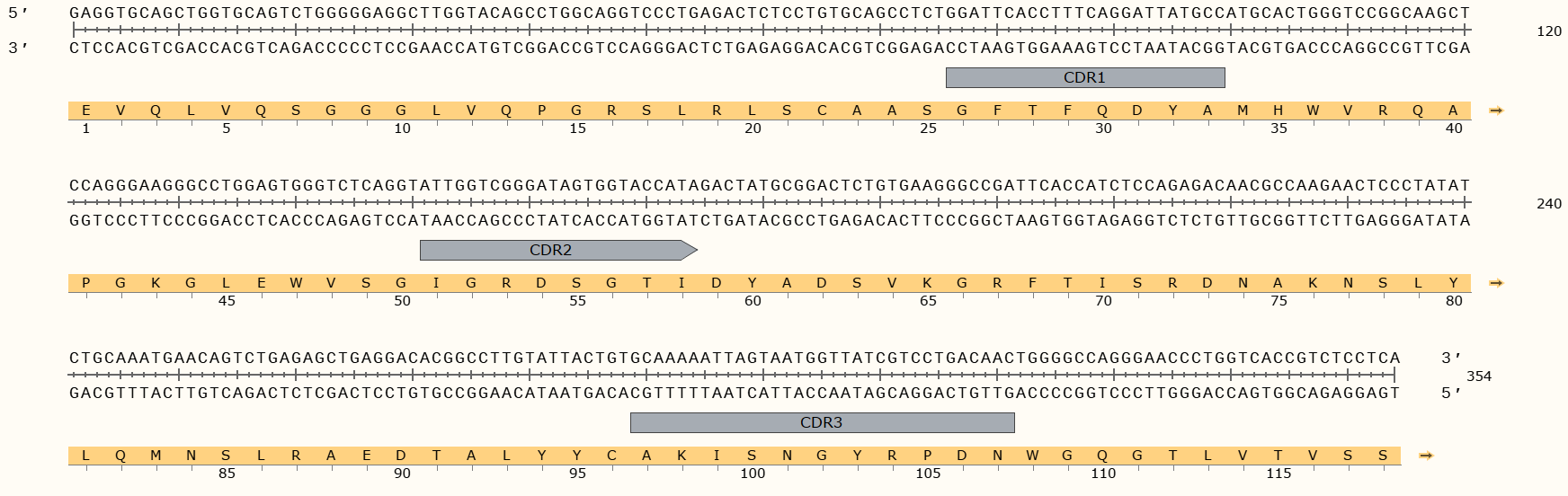** |
|  |

| **Light chain** |
| --- |
| **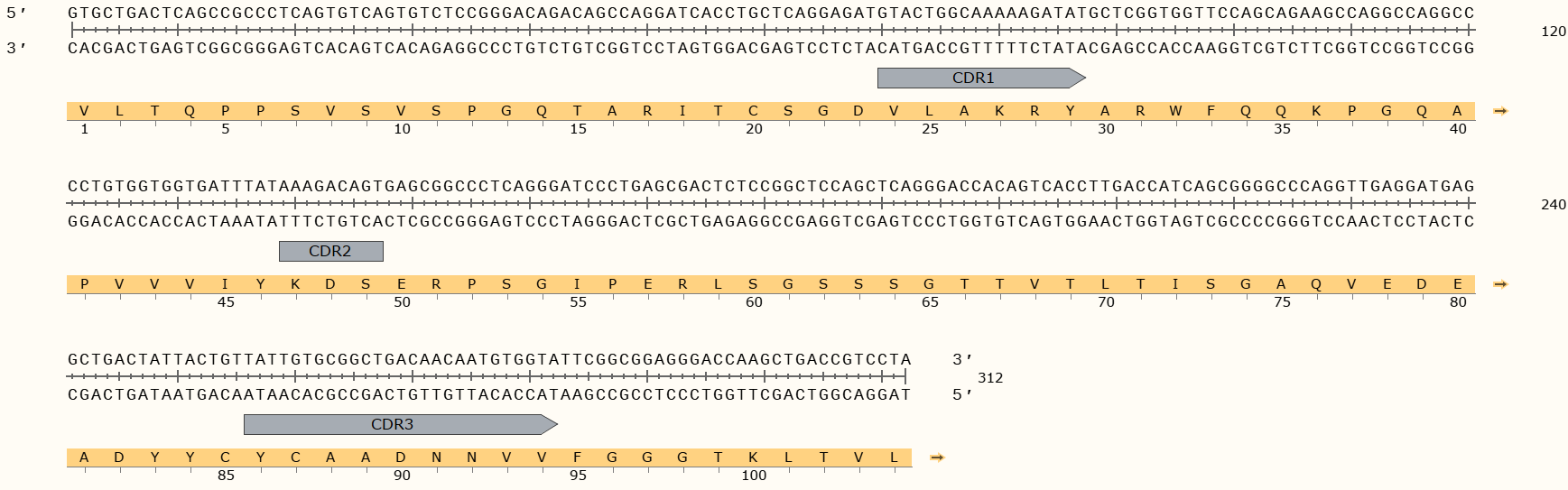** |

**D8 antibody**

| **Heavy Chain** |
| --- |
| **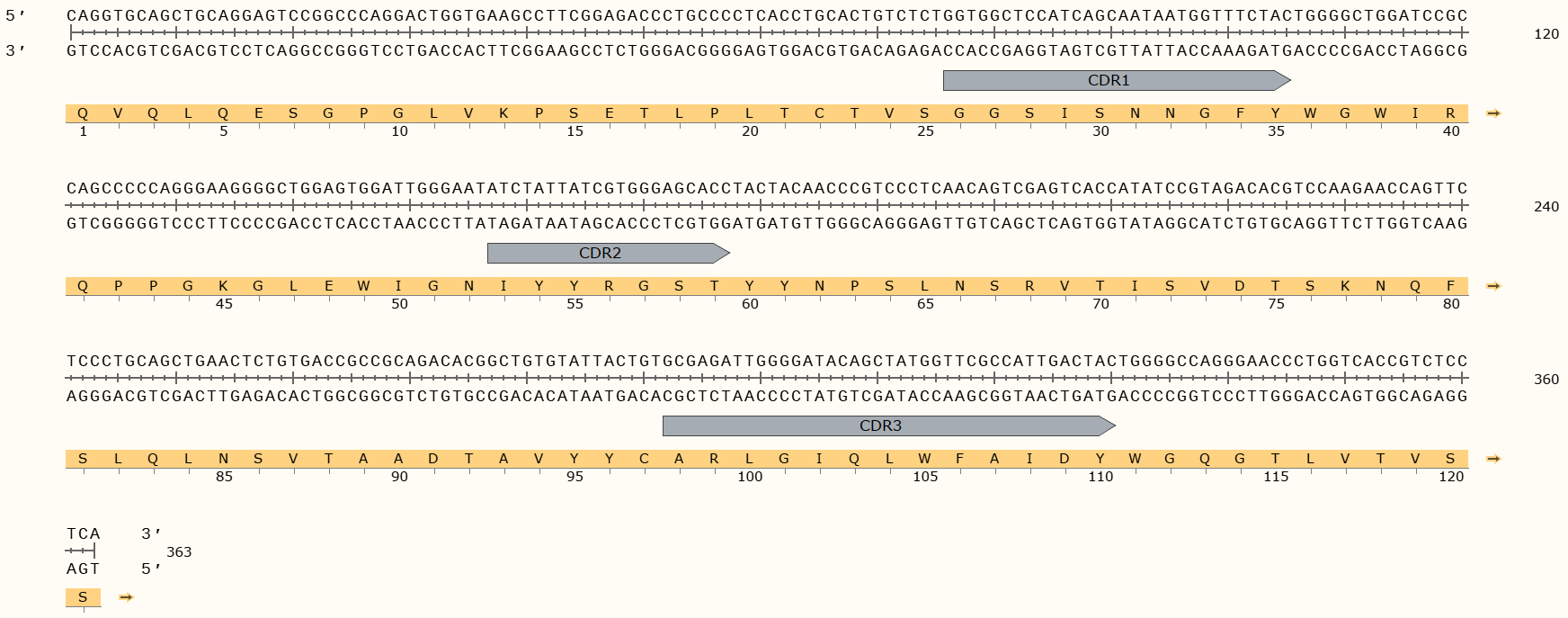** |
|  |
| **Light chain** |
| **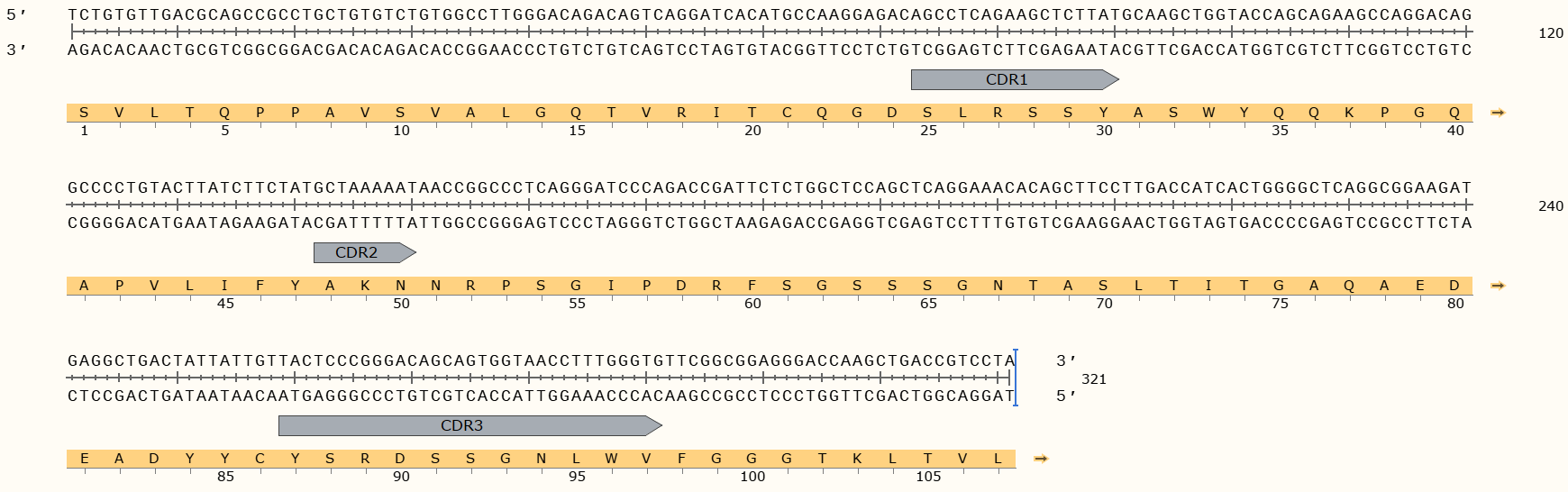** |
