# Supplementary material for: A nonstructural protein 1 capture enzyme-linked immunosorbent assay specific for dengue viruses
Source: PLoS One. 2023 May 18;18(5):e0285878. doi: 10.1371/journal.pone.0285878 (PMC10194908; doi:10.1371/journal.pone.0285878)
Supplement: S1 Raw images — These blots and gel images were cropped, compiled, and labeled to generate Fig 1B. (PDF) [file pone.0285878.s003.pdf]

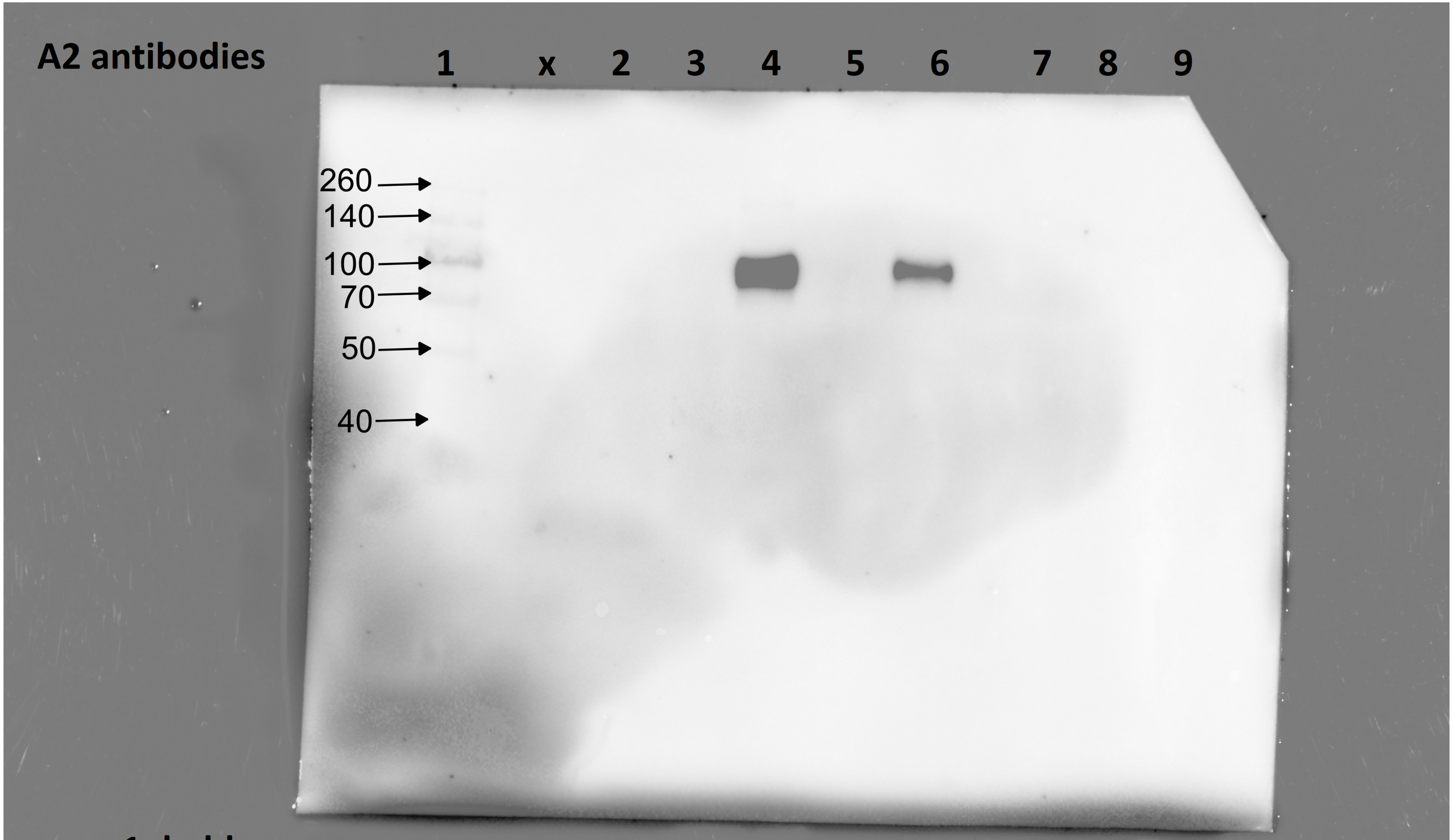

1. ladder
2. DENV1-NS1, no boil
3. DENV1-NS1, boil
4. DENV2-NS1, no boil
5. DENV2-NS1, boil
6. DENV3-NS1, no boil
7. DENV3-NS1, boil
8. DENV4-NS1, no boil
9. DENV4-NS1, boil

CHO antibodies

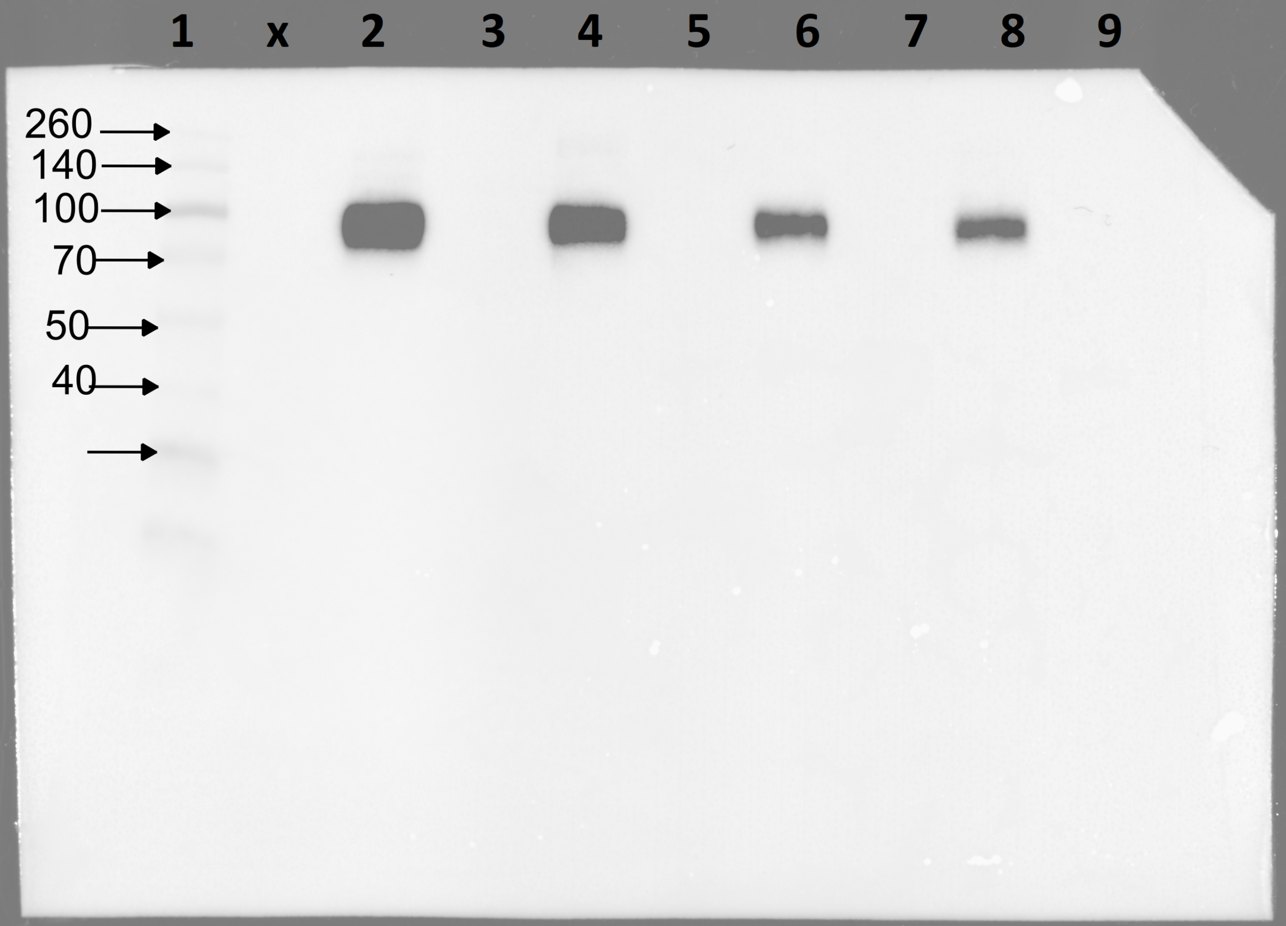

1. ladder
2. DENV1-NS1, no boil
3. DENV1-NS1, boil
4. DENV2-NS1, no boil
5. DENV2-NS1. boil
6. DENV3-NS1, no boil
7. DENV3-NS1, boil
8. DENV4-NS1, no boil
9. DENV4-NS1, boil

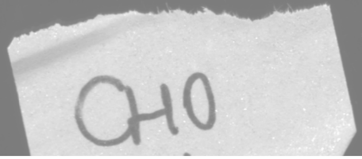

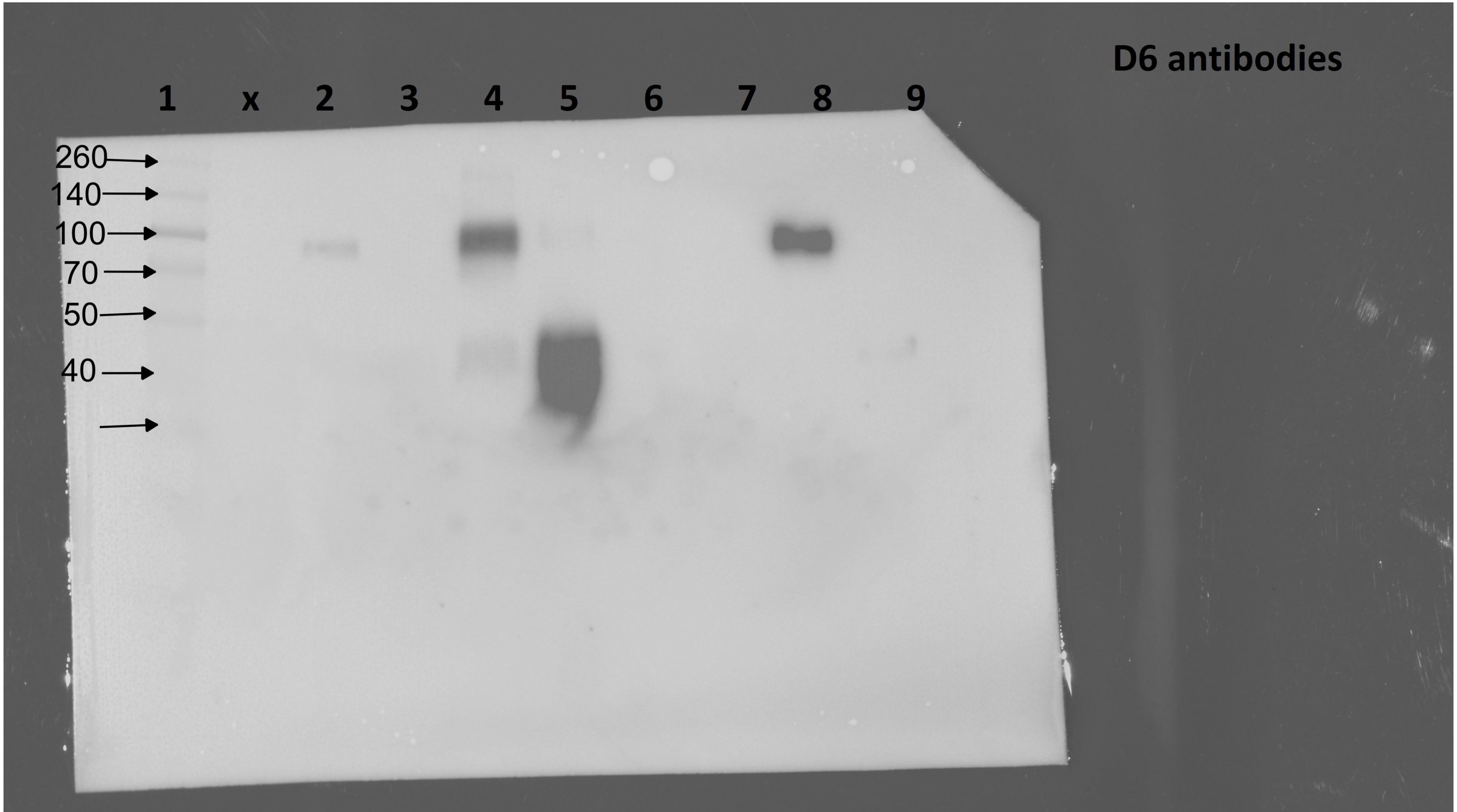

1. ladder
2. DENV1-NS1, no boil
3. DENV1-NS1, boil
4. DENV2-NS1, no boil
5. DENV2-NS1. boil
6. DENV3-NS1, no boil
7. DENV3-NS1, boil
8. DENV4-NS1, no boil
9. DENV4-NS1, boil

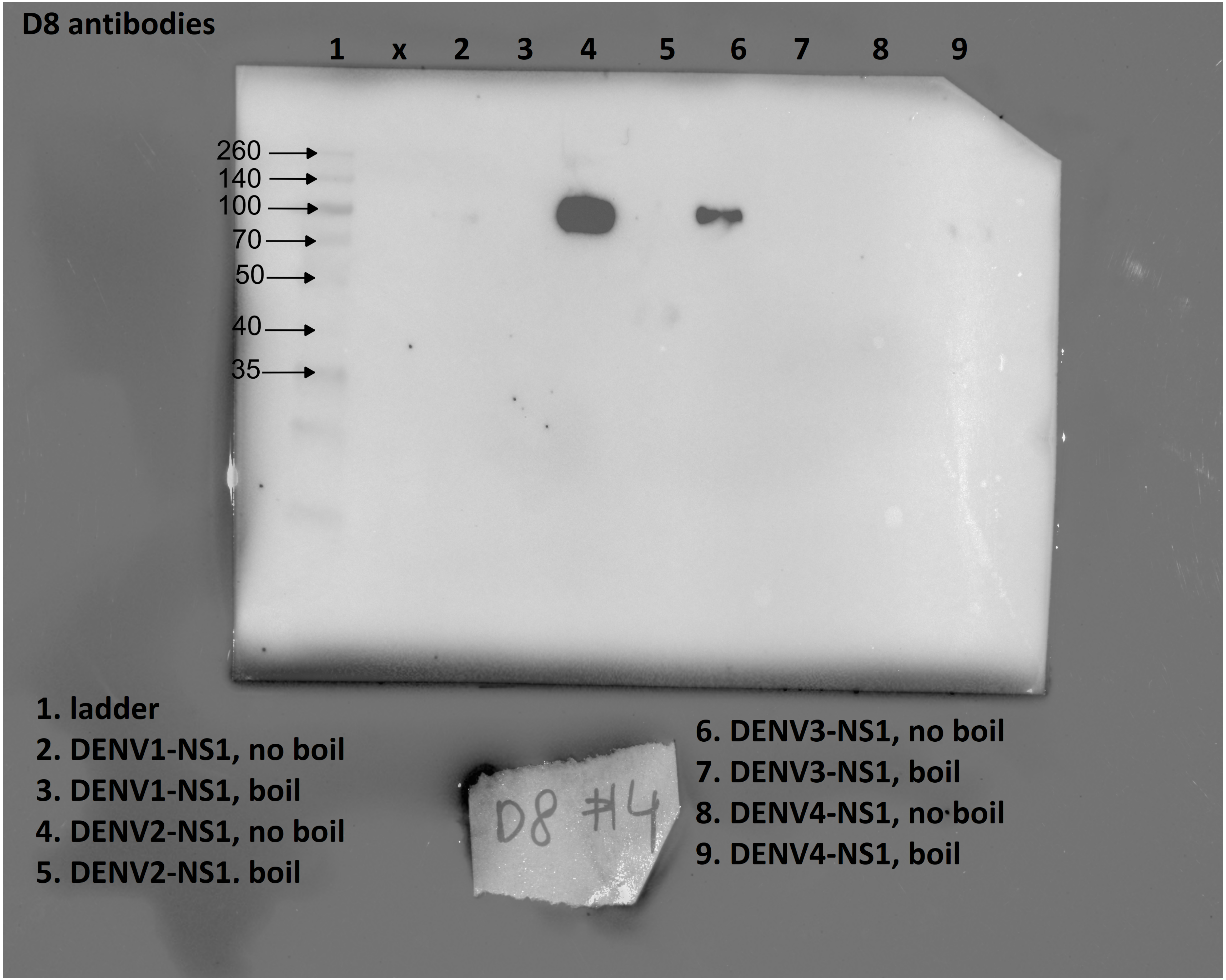
D8 antibodies
1 x 2 3 4 5 6 7 8 9
260
140
100
70
50
40
35
1. ladder
2. DENV1-NS1, no boil
3. DENV1-NS1, boil
4. DENV2-NS1, no boil
5. DENV2-NS1. boil
D8 #14
6. DENV3-NS1, no boil
7. DENV3-NS1, boil
8. DENV4-NS1, no boil
9. DENV4-NS1, boil

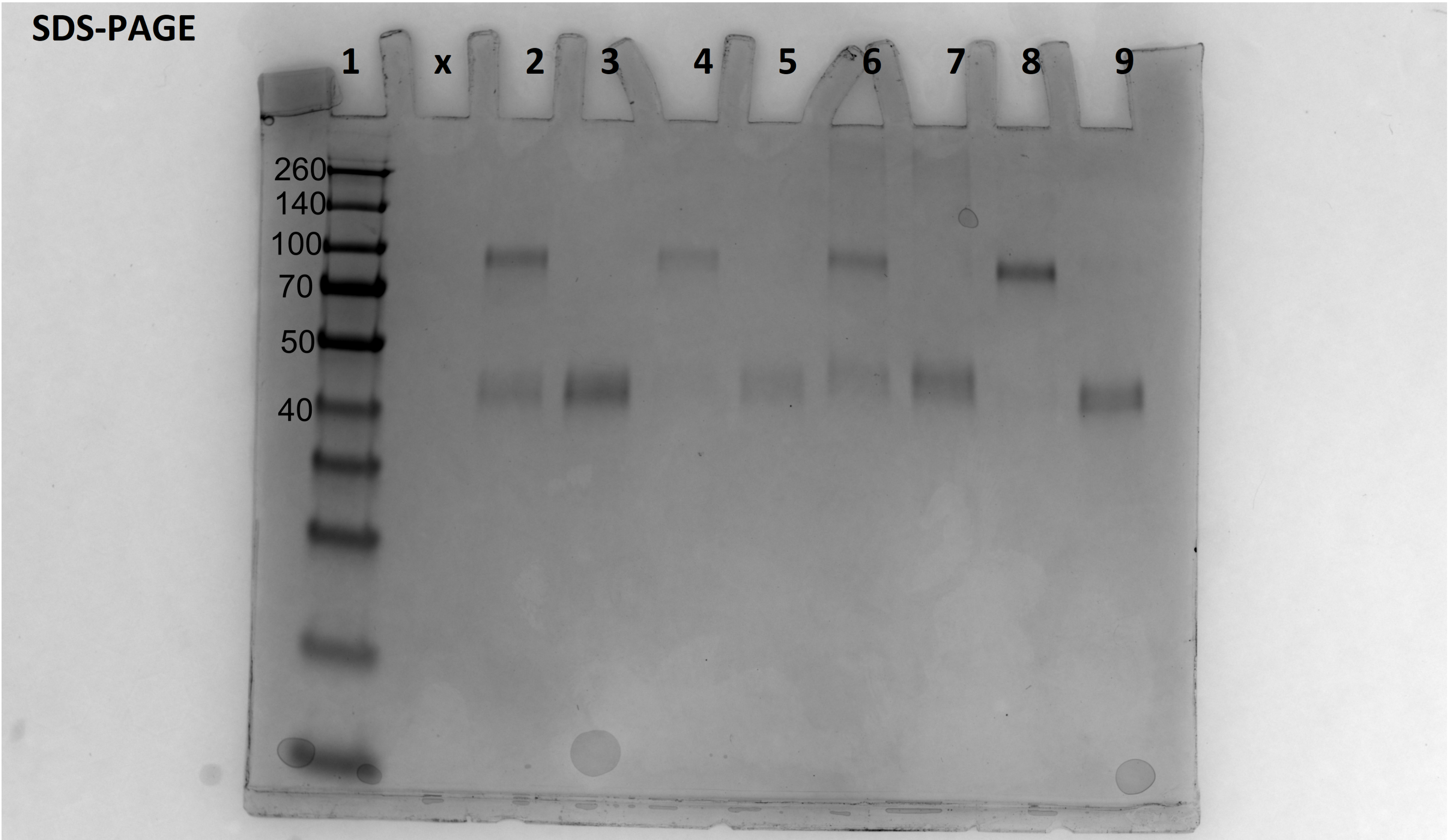

1. ladder
2. DENV1-NS1, no boil
3. DENV1-NS1, boil
4. DENV2-NS1, no boil
5. DENV2-NS1. boil
6. DENV3-NS1, no boil
7. DENV3-NS1, boil
8. DENV4-NS1, no boil
9. DENV4-NS1, boil
